# Supplementary material for: Impact of PEG sensitization on the efficacy of PEG hydrogel-mediated tissue engineering
Source: Nat Commun. 2024 Apr 18;15:3283. doi: 10.1038/s41467-024-46327-3 (PMC11026400; doi:10.1038/s41467-024-46327-3)
Supplement: Supplementary file 1 — Supplementary Information [file 41467_2024_46327_MOESM1_ESM.pdf]

SUPPLEMENTARY INFORMATION

Impact of PEG Sensitization on the Efficacy of PEG Hydrogel-Mediated Tissue Engineering

Alisa H. Isaac[1,2,3], Sarea Y. Recalde Phillips[1], Elizabeth Ruben[1], Matthew Estes[1], Varsha Rajavel[1], Talia Baig[1], Carol Paleti[4], Kirsten Landsgaard[1], Ryang Hwa Lee[4], Teja Guda[2,3], Michael F. Criscitiello[5], Carl Gregory[1,4], and Daniel L. Alge[1,6*]

[1]Department of Biomedical Engineering, Texas A&M University, College Station, TX, USA 77843
[2]Department of Biomedical Engineering and Chemical Engineering, The University of Texas at San Antonio, San Antonio, TX, USA 78249
[3]Department of Cell Systems and Anatomy, The University of Texas Health San Antonio, San Antonio, TX, USA 78229
[4]Department of Cell Biology and Genetics, School of Medicine, Texas A&M University, College Station, TX, USA 77843
[5]Comparative Immunogenetics Laboratory, Department of Veterinary Pathobiology, Texas A&M University, College Station, TX, USA 77843
[6]Department of Materials Science and Engineering, Texas A&M University, College Station, TX, USA 77843

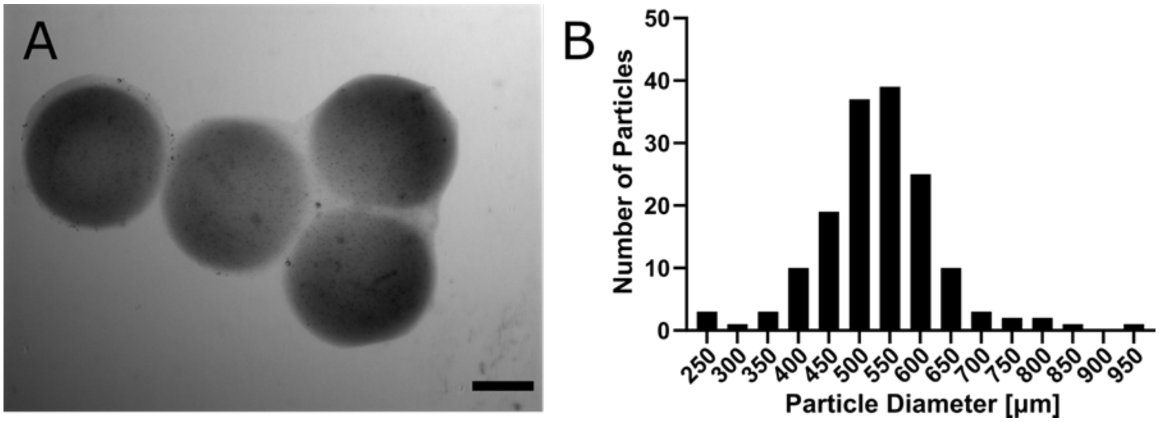

Supplementary Figure 1: Characterization of electrosprayed microgels. (A) Brightfield image of microgels stained with Trypan Blue (scale bar = 200µm). (B) Size distribution of microgels (n = 156).

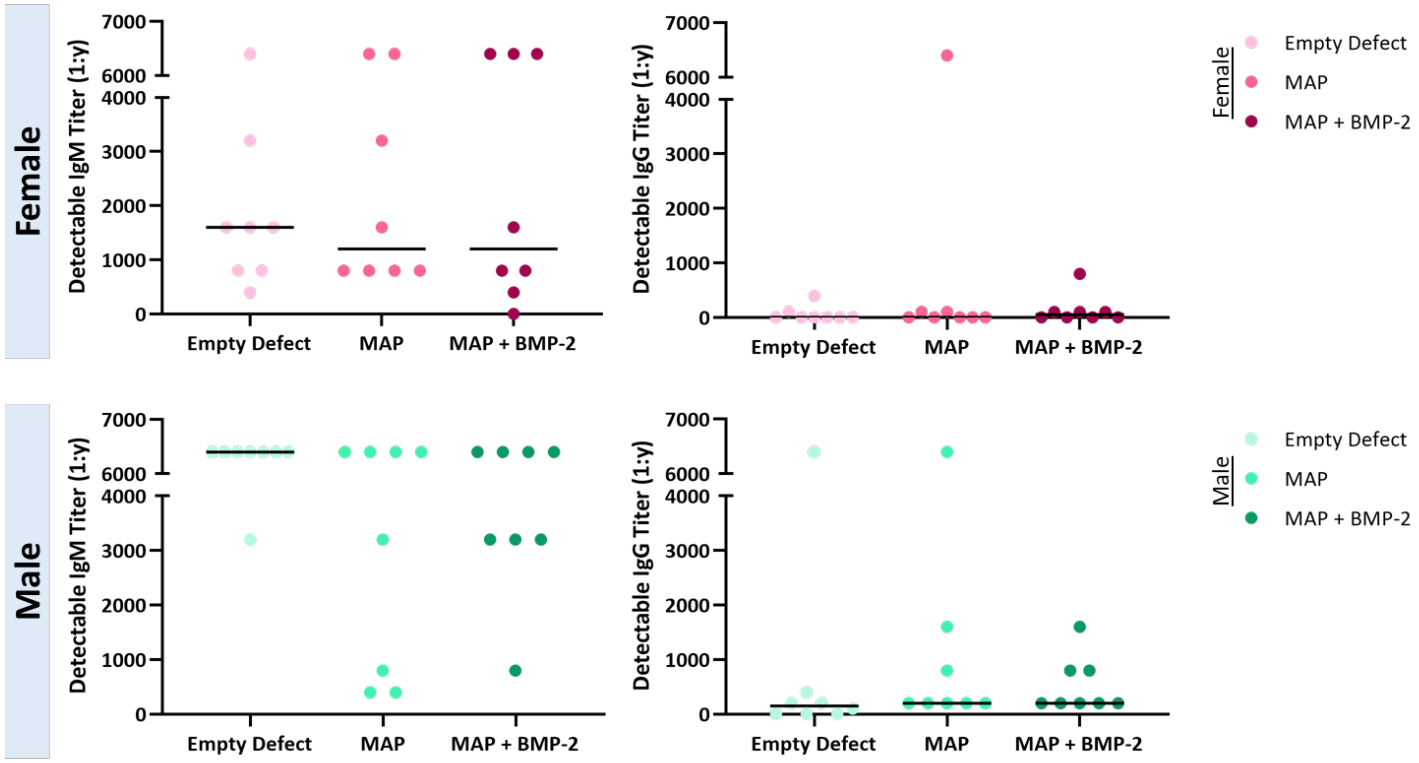

Supplementary Figure 2: Titers of anti-PEG IgM and IgG antibodies in female and male mice following PEG-KLH sensitization (n = 8 mice per treatment group). Dots represent individual specimens, and the black bars represent the median of the sample groups.

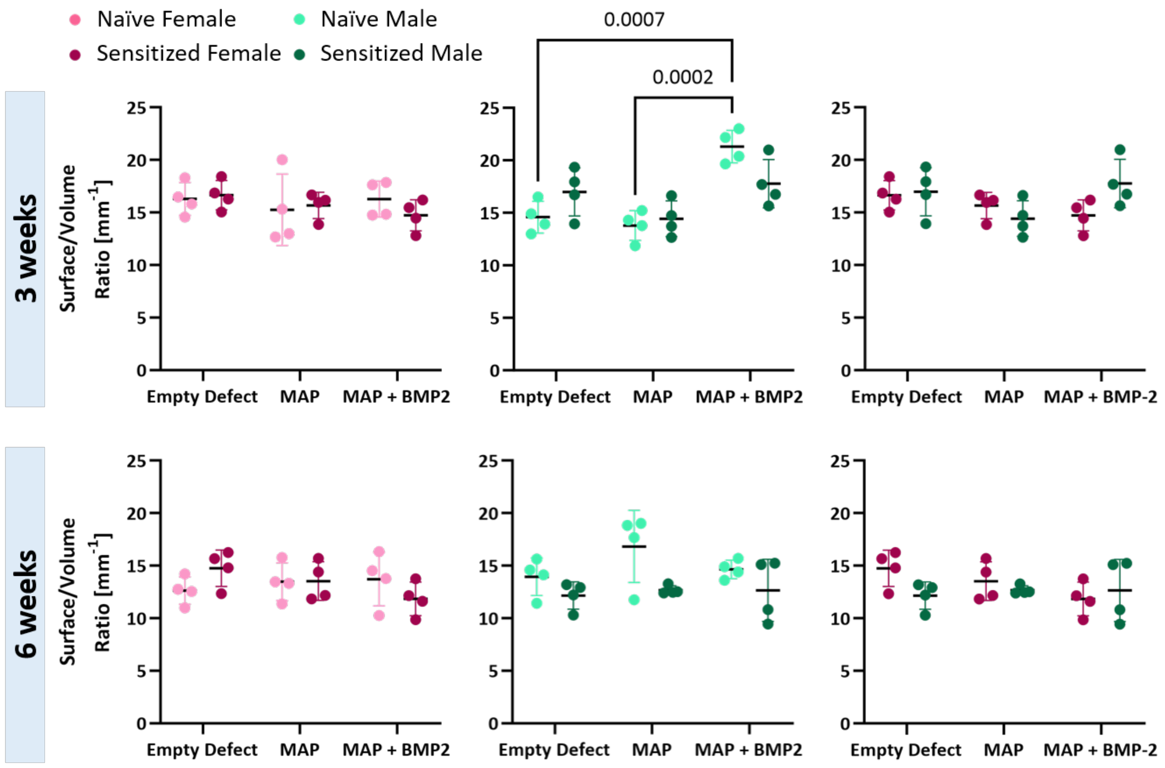

Supplementary Figure 3: Surface area-to-volume ratio of calvarial defects at 3 and 6 weeks post-implantation (n = 4 mice per treatment group). Statistical significance was calculated by ordinary two-way ANOVA with Tukey's multiple comparisons test with a single pooled variance, an alpha threshold of 0.05, and 95% confidence interval. Data is represented as mean ± SD, and dots represent individual specimens.

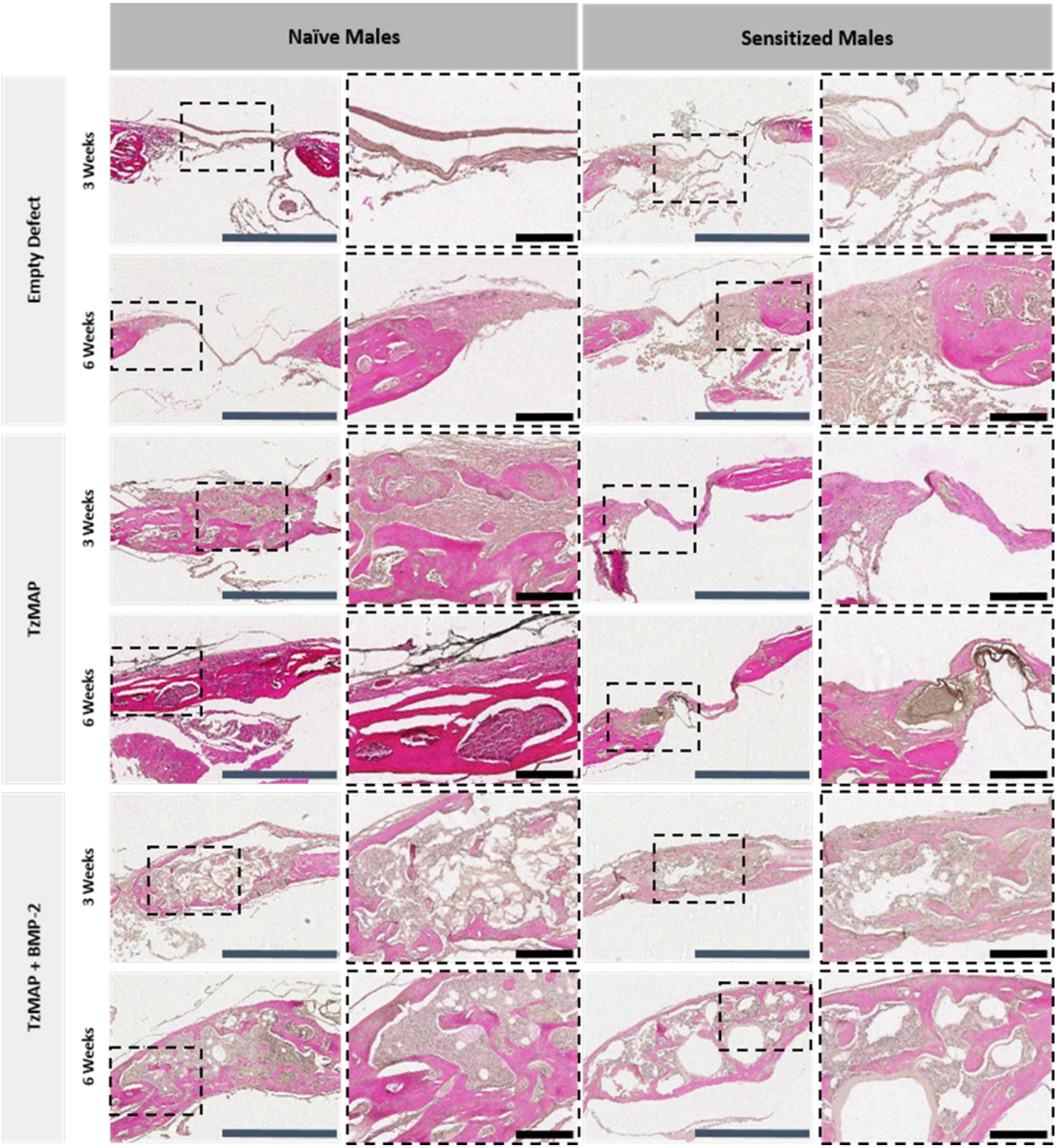

Supplementary Figure 4: H&E staining of calvarial defects of naïve (left) and sensitized (right) male mice left empty, treated with MAP, or treated with MAP + BMP-2 at 3 and 6 weeks post-implantation. The 10x images

on the right (scale bar = 200μm) are a magnified image of the dashed box of the 4x images (scale bar = 1mm). Images are representative of n = 4 mice for each treatment group.

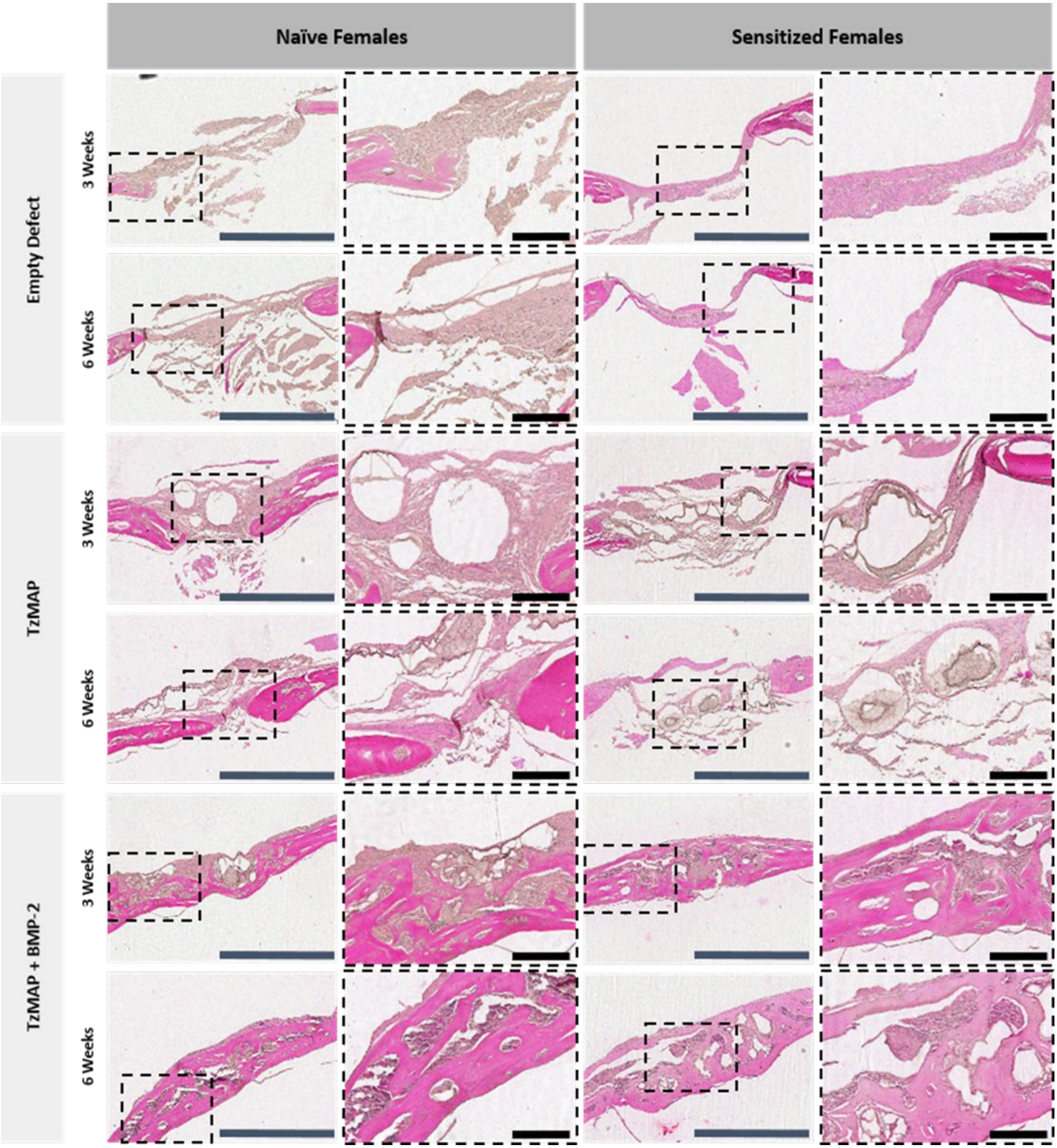

Supplementary Figure 5: H&E staining of calvarial defects of naïve (left) and sensitized (right) female mice left empty, treated with MAP, or treated with MAP + BMP-2 at 3 and 6 weeks post-implantation. The 10x images on the right (scale bar = 200μm) are a magnified image of the dashed box of the 4x images (scale bar = 1mm). Images are representative of n = 4 mice for each treatment group.

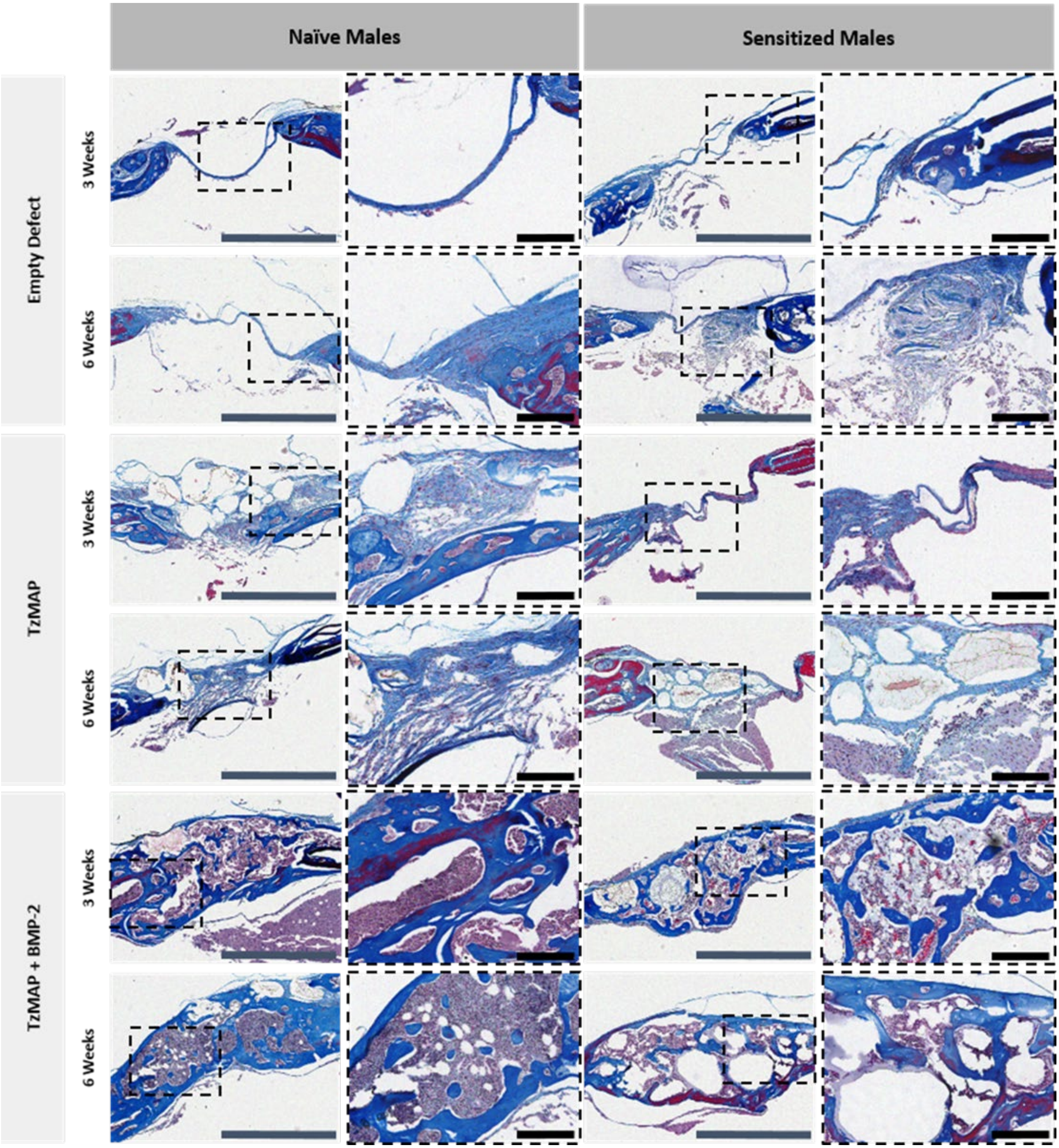

Supplementary Figure 6: Masson's trichrome staining of calvarial defects of naïve (left) and sensitized (right) male mice left empty, treated with MAP, or treated with MAP + BMP-2 at 3- and 6-weeks post-implantation. The 10x images on the right (scale bar = 200µm) is a magnified image of the dashed box of the 4x images (scale bar = 1mm). Images are representative of n = 4 mice for each treatment group.

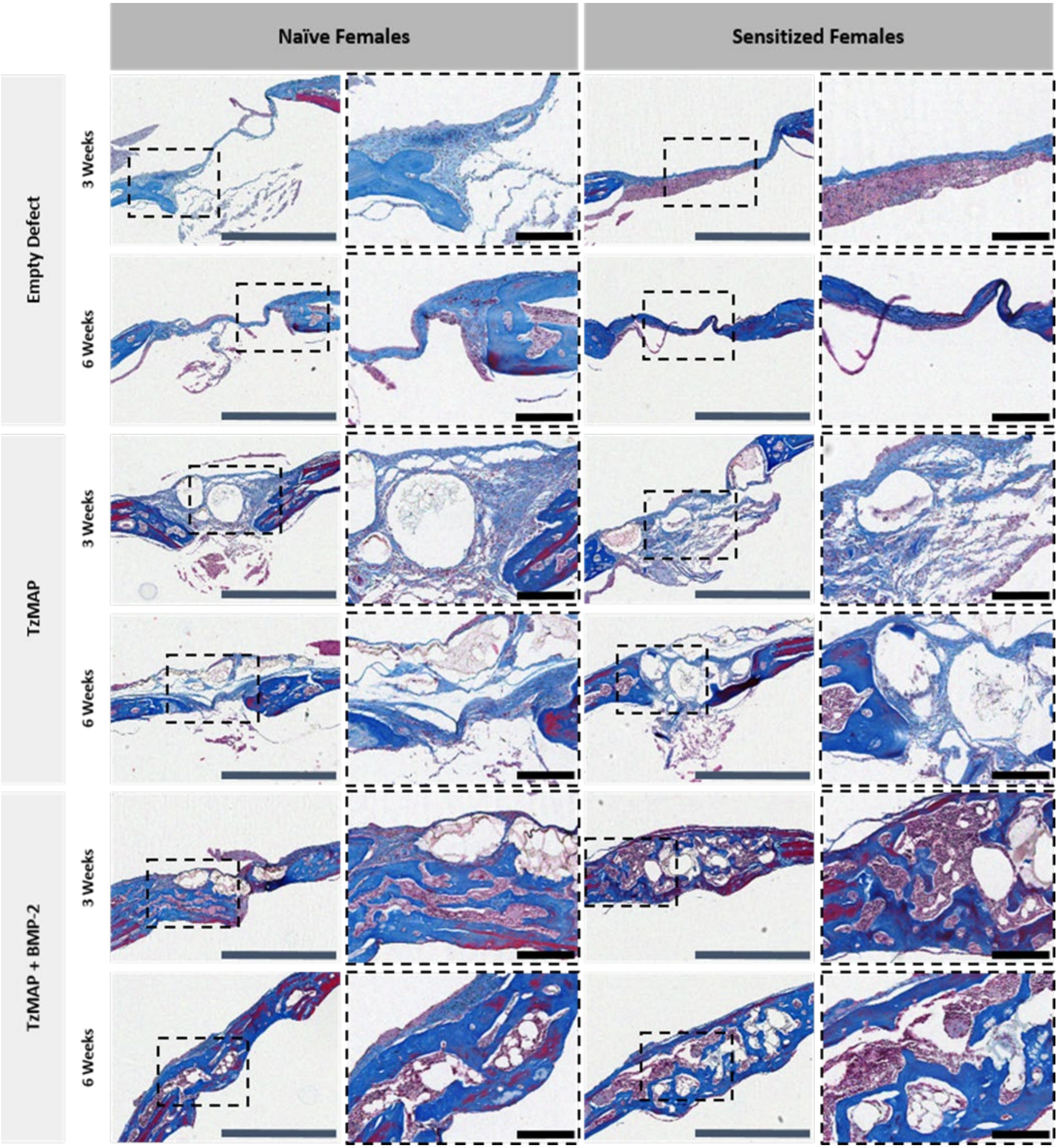

Supplementary Figure 7: Masson's trichrome staining of calvarial defects of naïve (left) and sensitized (right) female mice left empty, treated with MAP, or treated with MAP + BMP-2 at 3- and 6-weeks post-implantation. The 10x images on the right (scale bar = 200μm) is a magnified image of the dashed box of the 4x images (scale bar = 1mm). Images are representative of n = 4 mice for each treatment group.

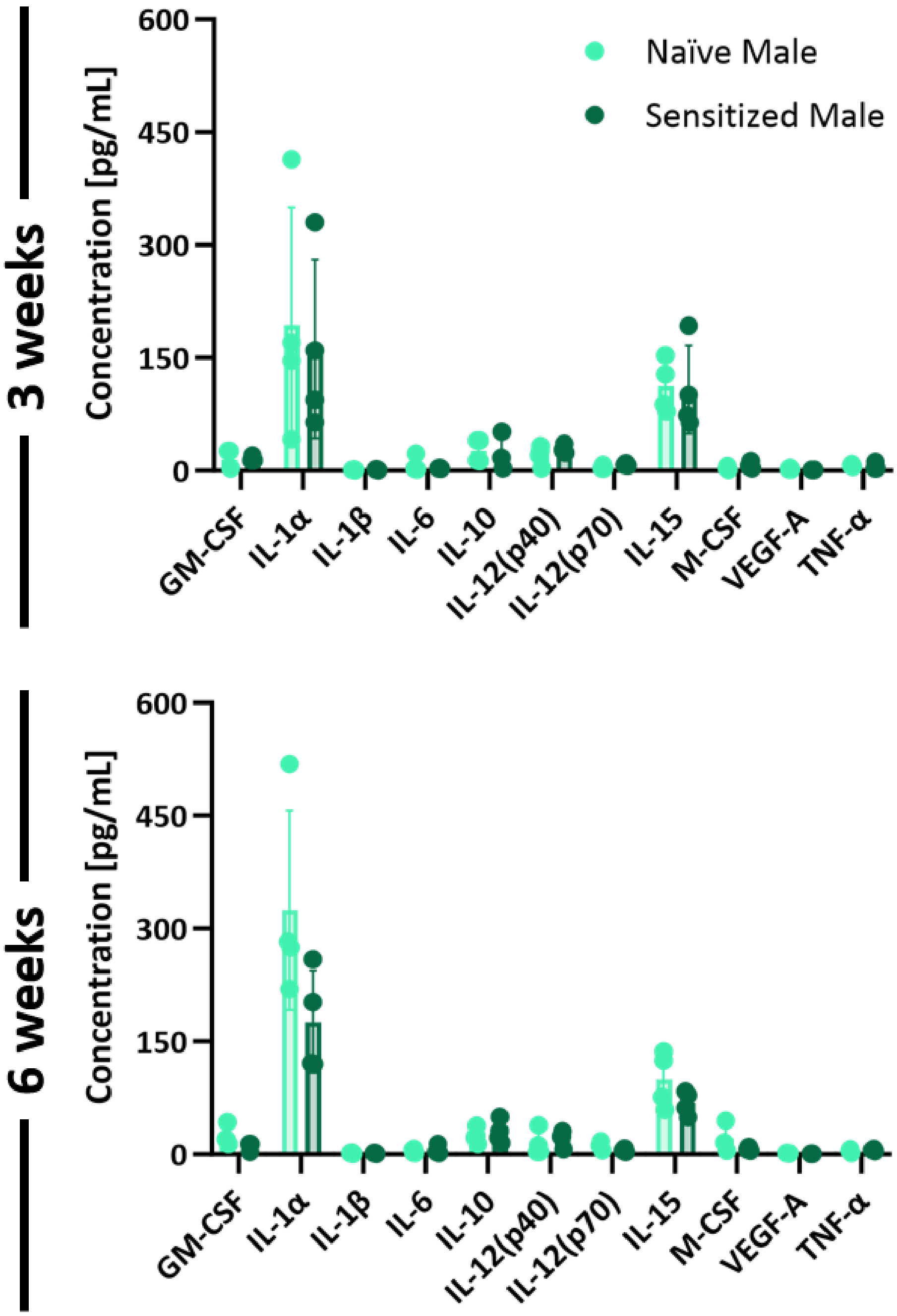

Supplementary Figure 8: Characterization of cytokine production at 3 and 6 weeks post-implantation for the MAP + BMP-2 treatment (n = 4 mice per treatment group). Statistical significance was calculated by multiple unpaired t test with Welch correction and False Discovery Rate approach using a two-stage linear step-up procedure of Benjamini Krieger and Yekutieli. Data is represented as mean ± SD, and dots represent individual specimens.
