# Supplementary material for: Impact of PEG sensitization on the efficacy of PEG hydrogel-mediated tissue engineering
Source: Nat Commun. 2024 Apr 18;15:3283. doi: 10.1038/s41467-024-46327-3 (PMC11026400; doi:10.1038/s41467-024-46327-3)
Supplement: Supplementary file 2 — Reporting Summary [file 41467_2024_46327_MOESM2_ESM.pdf]

nature portfolio

Corresponding author(s): Daniel Alge

Last updated by author(s): 2/6/2024

Reporting Summary

Nature Portfolio wishes to improve the reproducibility of the work that we publish. This form provides structure for consistency and transparency in reporting. For further information on Nature Portfolio policies, see our Editorial Policies and the Editorial Policy Checklist.

Statistics

For all statistical analyses, confirm that the following items are present in the figure legend, table legend, main text, or Methods section.

n/a	Confirmed	
☐	☒	The exact sample size (*n*) for each experimental group/condition, given as a discrete number and unit of measurement
☐	☒	A statement on whether measurements were taken from distinct samples or whether the same sample was measured repeatedly
☐	☒	The statistical test(s) used AND whether they are one- or two-sided<br>*Only common tests should be described solely by name; describe more complex techniques in the Methods section.*
☒	☐	A description of all covariates tested
☐	☒	A description of any assumptions or corrections, such as tests of normality and adjustment for multiple comparisons
☐	☒	A full description of the statistical parameters including central tendency (e.g. means) or other basic estimates (e.g. regression coefficient) AND variation (e.g. standard deviation) or associated estimates of uncertainty (e.g. confidence intervals)
☐	☒	For null hypothesis testing, the test statistic (e.g. *F*, *t*, *r*) with confidence intervals, effect sizes, degrees of freedom and *P* value noted<br>*Give P values as exact values whenever suitable.*
☒	☐	For Bayesian analysis, information on the choice of priors and Markov chain Monte Carlo settings
☒	☐	For hierarchical and complex designs, identification of the appropriate level for tests and full reporting of outcomes
☒	☐	Estimates of effect sizes (e.g. Cohen's *d*, Pearson's *r*), indicating how they were calculated

Our web collection on statistics for biologists contains articles on many of the points above.

Software and code

Policy information about availability of computer code

Data collection	MicroCT data was collected on a Bruker Skyscan 1275 using software version 1.0.15. Motic Easy Scan (version 1.0.5.134) was used for imaging tissue slides.
Data analysis	MicroCT data was reconstructed using NRecon (version 1.7.4.6) and analyzed using CTAn (version 1.16.4.1). ImageJ (version 1.54d) was used to quantify collagen deposition. Statistical analysis was performed using GraphPad (version 10.1.2).

For manuscripts utilizing custom algorithms or software that are central to the research but not yet described in published literature, software must be made available to editors and reviewers. We strongly encourage code deposition in a community repository (e.g. GitHub). See the Nature Portfolio guidelines for submitting code & software for further information.

Data

Policy information about availability of data

All manuscripts must include a data availability statement. This statement should provide the following information, where applicable:

- Accession codes, unique identifiers, or web links for publicly available datasets
- A description of any restrictions on data availability
- For clinical datasets or third party data, please ensure that the statement adheres to our policy

All data needed to evaluate the conclusions in the paper are present in the paper and/or the Supplementary Materials. Source data are provided with this paper and are available online in the Figshare repository (DOI: https://doi.org/10.6084/m9.figshare.24769155).

Research involving human participants, their data, or biological material

Policy information about studies with human participants or human data. See also policy information about sex, gender (identity/presentation), and sexual orientation and race, ethnicity and racism.

Reporting on sex and gender	N/A
Reporting on race, ethnicity, or other socially relevant groupings	N/A
Population characteristics	N/A
Recruitment	N/A
Ethics oversight	N/A

Note that full information on the approval of the study protocol must also be provided in the manuscript.

Field-specific reporting

Please select the one below that is the best fit for your research. If you are not sure, read the appropriate sections before making your selection.

[x] Life sciences [ ] Behavioural & social sciences [ ] Ecological, evolutionary & environmental sciences

For a reference copy of the document with all sections, see nature.com/documents/nr-reporting-summary-flat.pdf

Life sciences study design

All studies must disclose on these points even when the disclosure is negative.

Sample size	A sample size of N=4 was chosen based on a power analysis and data we previously published (DOI: 10.1126/scitranslmed.3003396). Based on this power analysis, N=4 was deemed sufficient for detecting a 30% effect size with 20% standard deviation at alpha = 0.05 and 80% power.
Data exclusions	No data were excluded from the analyses.
Replication	The PEG sensitization protocol using subcutaneous injections of PEG-KLH has been replicated eight times independently with similar results. The calvarial defect study was performed once using independent biological quadruplicates but was not otherwise replicated due to funding constraints.
Randomization	All mice for this study were caged in groups of 4 upon receipt. Half of the cages were allocated to the PEG sensitization group and half were allocated to the control group. They were all the same age at the start of the study and received injections of PEG-KLH or saline on the same schedule, and the same schedule for surgery was followed.
Blinding	Mice were assigned ID codes that blinded the investigators to the sample groups during data collection and analysis.

Reporting for specific materials, systems and methods

We require information from authors about some types of materials, experimental systems and methods used in many studies. Here, indicate whether each material, system or method listed is relevant to your study. If you are not sure if a list item applies to your research, read the appropriate section before selecting a response.

Materials & experimental systems

n/a	Involved in the study	
[ ]	[x]	Antibodies
[x]	[ ]	Eukaryotic cell lines
[x]	[ ]	Palaeontology and archaeology
[ ]	[x]	Animals and other organisms
[x]	[ ]	Clinical data
[x]	[ ]	Dual use research of concern
[x]	[ ]	Plants

Methods

n/a	Involved in the study	
[x]	[ ]	ChIP-seq
[x]	[ ]	Flow cytometry
[x]	[ ]	MRI-based neuroimaging

Antibodies

Antibodies used	HRP conjugated Goat anti-Mouse IgG Fc (Invitrogen, catalog # A16084, lot # 93-40-080422), HRP conjugated Goat anti-Mouse IgM (Heavy Chain) (Invitrogen, catalog # 62-6820, lot # YB372990), Rabbit anti-Mouse/Rat/Human CD3 (Abcam, catalog # ab5690, lot #

YB372990), Rabbit anti-Mouse/Rat CD68 (Invitrogen, catalog # PA5-78996, lot # YB372990), HRP conjugated Goat Anti-Rabbit IgG H&L (Abcam, catalog # ab205718, lot # YB372990). All antibodies are polyclonal.

Validation

According to the Invitrogen website, HRP conjugated Goat anti-Mouse IgG Fc (catalog # A16084; https://www.thermofisher.com/antibody/product/Goat-anti-Mouse-IgG-Fc-Secondary-Antibody-Polyclonal/A16084) and HRP conjugated Goat anti-Mouse IgM (Heavy Chain) (catalog # 62-6820, https://www.thermofisher.com/antibody/product/Goat-anti-Mouse-IgM-Heavy-chain-Secondary-Antibody-Polyclonal/62-6820) are both validated for use in ELISA, and Rabbit anti-Mouse/Rat CD68 (Invitrogen, catalog # PA5-78996, https://www.thermofisher.com/antibody/product/CD68-Antibody-Polyclonal/PA5-78996) is validated for use in immunohistochemistry.

According to the Abcam website, Rabbit anti-Mouse/Rat/Human CD3 (Abcam, catalog # ab5690, https://www.abcam.com/products/primary-antibodies/cd3-epsilon-antibody-ab5690.html) and HRP conjugated Goat Anti-Rabbit IgG H&L (Abcam, catalog # ab205718, https://www.abcam.com/products/secondary-antibodies/goat-rabbit-igg-hl-hrp-ab205718.html) are both validated for use in immunohistochemistry.

Lists of publications in which these antibodies have been used are also provided on the respective websites.

Animals and other research organisms

Policy information about studies involving animals; ARRIVE guidelines recommended for reporting animal research, and Sex and Gender in Research

Laboratory animals

8 week old C57BL/6 mice were used in this study.

Wild animals

No wild animals were used in this study.

Reporting on sex

N=4 males and N=4 females were used in this study. Data for males and females is disaggregated in the manuscript and source data, and sex-dependent differences are noted in the manuscript.

Field-collected samples

No field collected samples were used in this study.

Ethics oversight

This study was approved by the Texas A&M Institutional Animal Care and Use Committee under protocol 2018-0134. This is stated in the manuscript.

Note that full information on the approval of the study protocol must also be provided in the manuscript.

Plants

Seed stocks

N/A

Novel plant genotypes

N/A

Authentication

N/A
